# Cancer and pulmonary hypertension: Learning lessons and real-life interplay

**DOI:** 10.21542/gcsp.2020.10

**Published:** 2020-04-30

**Authors:** Soni Savai Pullamsetti, Sreenath Nayakanti, Prakash Chelladurai, Argen Mamazhakypov, Siavash Mansouri, Rajkumar Savai, Werner Seeger

**Affiliations:** 1 Max Planck Institute for Heart and Lung Research, Member of the German Center for Lung Research (DZL), Member of the Cardio-Pulmonary Institute (CPI), Bad Nauheim, 61231, Germany; 2 Department of Internal Medicine, Member of the DZL, Member of CPI, Justus Liebig University, Giessen, 35392, Germany; 3 Institute for Lung Health (ILH), Member of the DZL, Justus Liebig University, Giessen, 35392, Germany

## Abstract

This article reviews the scientific reasons that support the intriguing vision of pulmonary hypertension (PH) as a disease with a cancer-like nature and to understand whether this point of view may have fruitful consequences for the overall management of PH. This review compares cancer and PH in view of Hanahan and Weinberg’s principles (i.e., hallmarks of cancer) with an emphasis on hyperproliferative, metabolic, and immune/inflammatory aspects of the disease. In addition, this review provides a perspective on the role of transcription factors and chromatin and epigenetic aberrations, besides genetics, as “common driving mechanisms” of PH hallmarks and the foreseeable use of transcription factor/epigenome targeting as multitarget approach against the hallmarks of PH. Thus, recognition of the widespread applicability and analogy of these concepts will increasingly affect the development of new means of PH treatment.

## Introduction

Pulmonary hypertension (PH) is a progressive disease of multifactorial etiology that has a poor prognosis and results in right heart failure. There are 5 classes of PH defined based on pathogenetic background and histopathological appearance, each of them encompassing several subclasses^1^. Class 1, pulmonary arterial hypertension (PAH), for which loss-of-function mutations in the transforming growth factor β (TGF-ß)/bone morphogenetic protein (BMP) superfamily have been identified as an underlying pathomechanism^2^, and idiopathic PAH (IPAH); class 2, PH due to left heart diseases; class 3, PH due to alveolar hypoxia and/or respiratory system disorders; class 4, PH due to (thrombo)embolic diseases; and class 5, PH due to a group of mixed diseases directly affecting pulmonary vessels. Taken together, all variants of PH affect up to 100 million people worldwide.

The vascular pathology of PH is characterized by pulmonary vasoconstriction and abnormal (“pseudomalignant”) inward remodeling processes, which may affect all vessel layers (intima, media, and adventitia)^3^. These remodeling processes result in severe loss of cross-sectional area and a concomitant increase in right ventricular (RV) afterload. Intimal changes include initial endothelial injury/apoptosis, endothelial cell (EC) proliferation, intimal invasion by (myo)fibroblast-like cells, enhanced matrix deposition, and, to a varying degree, vascular lumen obstruction by unique plexiform lesions.

A prominent feature in virtually all PH entities is vascular smooth muscle cell (SMC) proliferation, causing medial hypertrophy of the intra-acinar muscular resistance vessels and muscularization of the normally nonmuscularized precapillary arterioles. In several forms of PH, these structural abnormalities are accompanied by marked adventitia hypertrophy including (myo)fibroblast proliferation/invasion and enhanced matrix deposition. A further pathological feature is the accumulation of various inflammatory cells, particularly in the adventitia and media.

Overall, these structural changes suggest a switch from “quiescent” toward “pro-proliferative,” “apoptosis resistant,” and “pro-inflammatory” vascular cell phenotypes. As a functional consequence, pulmonary vascular resistance drastically increases, causing increased RV afterload with right heart hypertrophy and failure as further cardiac sequelae of the disease^3,4^.

The clinically observed imbalance of vasomotion (sustained vasoconstriction) in combination with altered production of endothelial vasoactive mediators, in particular nitric oxide (NO), prostacyclin (PGI2), and endothelin (ET), represented the starting point for the development of treatment options for PH in adults. These efforts resulted in 3 currently approved therapeutic modalities for the treatment of PAH: prostaglandin (PG) I2 and analogs (iloprost, treprostinil), phosphodiesterase (PDE) 5 inhibitors (sildenafil, tadalafil), and ET receptor antagonists (bosentan, ambrisentan; macitentan as novel development)^5^.

The first soluble guanylate cyclase stimulator (riociguat) has been proven to demonstrate efficacy in both PAH and chronic thromboembolic pulmonary hypertension (CTEPH)^6,7^ and has recently enriched the therapeutic armamentarium. Current PH therapies provide symptomatic relief and improve prognosis, but fall short in the restoration of structural and functional pulmonary vascular integrity, as a basis of long-term disability-free survival. Nevertheless, there are strong preclinical and clinical evidence showing that all these approaches not only affect the imbalanced pulmonary vasomotion but also possess some capacity to interfere with structural abnormalities characterizing PH.

However, we are far away from having a comprehensive molecular understanding of these remodeling processes and—even more—from a treatment concept to fully prevent or even reverse the maladaptive structural processes in the lung vasculature and RV under conditions of PH. Hence, it is important to decipher the molecular mechanisms behind maladaptive inward remodeling processes in the lung vasculature in PH to develop therapeutic approaches to prevent or reverse such processes.

A challenge in current PH research is the restoration of physiological vascular structure and function^5^, which represents the central therapeutic long-term goal. This aim requires better understanding of the switch from “quiescent” to “proliferative” cell phenotypes, disruption of the vicious pathogenic circuits that drive angioproliferative abnormalities, and activation of repair and regenerative mechanisms. Such major steps forward are a prerequisite for improving the persisting poor prognosis of PH patients. Accordingly, advances in the field of cancer provide unprecedented opportunities for a new understanding of PH and may offer new approaches for PH treatment.

It has become increasingly clear that PH can be viewed as a proliferative disease and has an incredible number of pathogenic mechanisms similar to cancer^5,8–11^. According to Hanahan and Weinberg, the vast majority of cancer hallmarks during disease progression (except tissue invasion and metastasis) are also shared by pulmonary vascular cells in PH patients. Importantly, key questions driving the field of cancer to understand how tumor cells reprogram to gain growth advantage, metabolism, and the contribution of surrounding immune/inflammatory microenvironment to benefit tumorigenesis are also applicable to PH. Recent experimental and conceptual advances in cancer cell metabolism, evading immune destruction and inflammation by innate immune cells, provide the field of PH with the unique opportunity to target metabolism and immune/inflammation axis.

**Figure 1.**
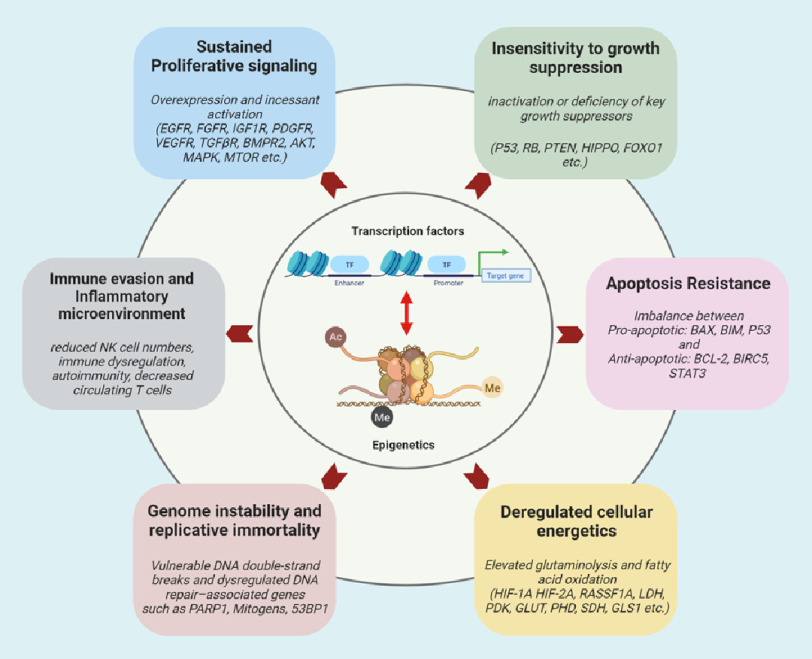
Hallmarks and underlying molecular signature of PH.

This article aims to review the scientific reasons that support the intriguing vision of PH as a disease with a cancer-like nature and to understand whether this point of view may have fruitful consequences for the overall management of PH. Hence, we compare cancer and PH in light of Hanahan and Weinberg’s principles (i.e., hallmarks of cancer) with an emphasis on hyperproliferative, metabolic, and immune/inflammatory aspects of the disease (Figure 1).

In addition, this review provides a perspective on the role of transcription factors and chromatin and epigenetic aberrations, besides genetics, as “common driving mechanisms” of PH hallmarks and the foreseeable use of transcription factor/epigenome targeting approach against the hallmarks of PH. We strongly believe that recognition of the widespread applicability and analogy of these concepts will increasingly affect the development of new means of PH treatment.

### I. Sustained proliferative signaling in PH

Sustained proliferative capacity is the central feature of many cancers and is mainly achieved by overexpression of receptor tyrosine kinases (EGFR, FGFR, IGF1R, PDGFR, and VEGFR) and their subsequent activation in an autocrine manner^11^. PDGF and PDGFR expression in lung tissues and pulmonary artery SMCs (PASMCs) isolated from PAH patients is significantly increased compared with healthy lungs^12^. In addition, PASMC-derived IGF-1 was shown to play a critical role in hypoxia-induced PH and pharmacological inhibition or genetic depletion of IGF-1 in SMC-attenuated hypoxia-induced remodeling in the lung vasculature of neonatal mice^13^.

On the other hand, both VEGF and VEGFR-2 are overexpressed in pulmonary artery ECs (PAECs) in plexiform lesions, suggesting that PAECs are stimulated in an autocrine manner by their own growth signals^14^.

EGFR overactivation by serine elastases is implicated in the pathobiology of PAH^15^. PAEC-derived FGF2 is also implicated in PAH development^16^. The binding of these signaling molecules to their receptors activates various downstream signaling molecules such as MEK/ERK, PI3K/AKT, and JAK/ STAT3 to induce cell growth, cell cycle progression, and cell survival^10^. Notably, pulmonary vascular cells isolated from PAH lungs also exhibit sustained hyperproliferative potential even under low or absence of growth factors^8,17^, suggesting constitutive activation of “cell intrinsic” mechanisms related to cell growth.

Receptor serine/threonine kinases transduce signals via the TGF-β and BMP and tumor-promoting arm of this family protein are often therapeutically targeted in many cancers. Similarly, heterozygous loss-of-function mutations in BMP receptor type II (BMPR2) and in ALK1, ENG, and BMP9 have been implicated in the pathogenesis of PAH. In another study, selective TGF-β1 and TGF-β3 blockade was helpful in reducing PH and pulmonary vascular remodeling in 3 rodent PH models, all of which exhibited excessive TGF-β signaling^18–20^.

### II. Insensitivity to growth suppression in PH

Like in cancers, pulmonary vascular cells in PH lungs have inactivation or deficiency of key growth suppressors, including p53, Rb, PTEN, Hippo, and FoxO1. Inactivation of these growth suppressors plays an important role in PASMC hyperproliferation, pulmonary vascular remodeling, and PH. p53 is a well-known tumor suppressor protein, and loss-of-function mutations of this gene are linked to human cancers. However, p53 knockout mice do not develop spontaneous PH, but develop severe PH under chronic hypoxia. Similarly, pharmacological p53 inhibition induces pulmonary vascular remodeling and aggravates monocrotaline-induced PH in rats^5^.

These data suggest that in contrast to cancers, p53 plays an important role in PH progression, rather than in disease initiation. Furthermore, the Rb pathway consists of 5 families of proteins: CDKN (e.g., Ink4a), D-type cyclins, cyclin-dependent protein kinases (cdk4, cdk6), the Rb family of pocket proteins (RB, p107, p130), and the E2F family of transcription factors^21^. Several components of this pathway, that is, p16Ink4a, cyclin D1, and RB, are frequently altered in cancer cells^22^.

Cyclin-dependent kinases (CDKs) are a family of serine-threonine kinases that control cell cycle and growth suppression^23^. In a recent study, CDKs were found to be overactivated and endogenous CDK inhibitors were underactivated in human PASMCs; therefore, using third-generation synthetic CDK4/6 inhibitors could reverse the proliferative phenotype of pulmonary vascular cells in various experimental PAH animal models^24^.

Phosphatase and tensin homologue (PTEN) is one of the highly mutated genes in many cancers. The activity of PTEN antagonizes the (PI3K)/AKT/mTOR pathway to repress tumor cell growth and survival^25^. Similar roles of PTEN have been implicated in PH pathogenesis; PTEN expression levels were significantly reduced in two preclinical PH models^26^. Notably, PTEN deletion in SMCs promoted widespread medial SMC hyperplasia and pulmonary vascular remodeling.

Mechanistically, PTEN inactivation was suggested to establish an autocrine growth loop and increase progenitor cell recruitment and subsequently PASMC hyperplasia through a HIF-1α-mediated SDF-1α/CXCR4 axis^27^. On the other hand, PTEN inactivation was also shown to promote PASMC hyperplasia through AKT-dependent HIF-1α upregulation^28,29^.

The Hippo signaling pathway is a growth control and tumor suppressor pathway that regulates cell proliferation. Defects in Hippo signaling and its downstream effectors Yes-associated protein (YAP) and transcriptional coactivator with PDZ-binding motif (TAZ) are associated with cancer development^30^. Hippo is inactivated in PASMCs in human PAH lungs and 2 PH rodent models^31^. Moreover, pharmacological inhibition of Hippo/LATS1/YAP pathway blocked apoptosis in pulmonary vascular cells induced by ACE2 activation^32^.

FoxOs are considered tumor suppressors that need to be inactivated to evade growth suppression by tumors. FoxO1 expression and activity were downregulated in human IPAH PASMCs and in 2 PH experimental models. Genetic of FoxO1 in vascular SMCs exhibited spontaneous PH phenotype, and either pharmacological reconstitution of FoxO1 activity with intravenous or inhaled paclitaxel or reconstitution of the transcriptional activity of FoxO1 by gene therapy reversed the PH phenotype both in vitro and in vivo, suggesting reconstitution of FoxO1 activity as a potential therapeutic option for PH^33^.

### III. Apoptosis resistance in PH

Apoptosis resistance of PH vascular cells is another feature that is shared with cancer cells. Resistance to apoptosis by PASMCs and PAECs in PAH is linked to an imbalance between pro-apoptotic (Bax, Bim, p53) and antiapoptotic (Bcl-2, Survivin, STAT3) proteins. Notch1 implicated in cancers also plays an important role in regulating PAEC apoptosis^34^. Protein-kinase AKT that mitigates apoptosis is constitutively upregulated in PASMCs in human IPAH lungs and is required for PAH progression^11^. Survivin is an inhibitor of apoptosis and inhibitors targeting survivin are often used in cancer therapy, and corroborating studies on PH showed that selective survivin inhibition reverses hypoxia-induced chronic PH^35^.

#### Future perspectives

In essence, several genes and pathways that regulate sustained proliferation and apoptosis resistance are shared between cancer and PH, thereby opening up broad avenues for nonvasodilator drugs. However, we are not sure whether these pathways are similarly contributing to PAH progression and development and other PH groups, and emerging evidence also suggests that like cancers, PH is a complex disease involving various cell types and, in most cases, individuals are asymptomatic until clinically important symptoms arise.

Despite progress, many current therapies still lack the feature to completely stop disease progression and this is mainly attributed to inter- and intra-PH heterogeneity. A comprehensive characterization of the disease, innovative models, and strong experimental PH rodent models need to be developed to improve the translatability of preclinical studies. As we speak of cancer paradigm in PH, there has been several cutting-edge technologies in cancer research to increase the efficacy of drugs (e.g., biocompatible hybrid nanoparticles)^36^ and to modify the disease using gene-editing approaches (e.g., CRISPR) that can be adopted to PH research.

Along these lines, several nonvasodilator and cancer drugs (e.g., imatinib) have significantly improved PVR, cardiac output, and 6-minute-walk distance (6MWD) in PAH patients^37,38^. It is critical to understand the underlying pathobiological mechanisms resulting in responsiveness and nonresponsiveness of these drugs as they can have life-saving effects. The risk of systemic side effects observed in several clinical trials involving broad multikinase inhibitors for PH treatment may be overcome by lung-selective delivery of drugs packed in nanoparticles or liposomes for controlled release. This approach can improve pulmonary efficacy even at low drug dosage. Nevertheless, a better understanding of similarities and dissimilarities between PH and cancer will hopefully lead to development of novel therapeutic strategies.

### IV. Inflammation and immunity in PH

Pulmonary vascular remodeling is associated with vascular wall infiltration with inflammatory and immune cells in PH lung tissues, in addition to augmented increase in pulmonary vascular cell proliferation. For example, the accumulation of diverse inflammatory and immune cell subpopulations, including CD4^+^ and CD8^+^ T cells, B cells, monocytes, macrophages, mast cells, and dendritic cells, within the remodeled pulmonary artery wall and perivascular regions has been established as an additional pathological feature of PAH^39,40^.

Importantly, it has been shown that marked perivascular inflammation observed in PAH lung tissues correlates with the degree of intima and media remodeling^41^. In addition, in PAH lung tissues, there is ectopic formation of tertiary lymphoid tissues localized in close proximity to the remodeled vessels consisting of various immune cells^42^.

In addition to IPAH, PAH may also develop because of various autoimmune diseases, such as systemic sclerosis (SSc), mixed connective tissue disease, and systemic lupus erythematosus^43^. The development of PAH in such autoimmune diseases are related to autoantibody-mediated PAEC apoptosis^44^.

Interestingly, accumulating evidence indicates that IPAH patients also display increased circulating autoantibody levels^45–48^. The mechanism of IPAH induction is also linked to autoantibody-induced PAEC cell apoptosis^49^, vascular SMC contraction^50^, and pulmonary fibroblast dysfunction^51^. A very good example of impaired or suppressed immune system in promoting pulmonary vascular remodeling is human immunodeficiency virus infection, which is associated with increased prevalence of PAH^52^. In addition to class 1 PH (PAH), the importance of inflammation and immunity has been shown in classes 2 (PH due to left heart diseases)^53^, 3 (PH due to hypoxia and lung diseases)^54^, and 4 (CTEPH)^55^ PH patients.

Upon the identification of accumulated inflammatory/immune cells within the pulmonary artery wall and increased circulating inflammatory mediators in PAH patients, subsequent mechanistic studies using *in vitro* and *in vivo* approaches have provided great insights into the role of inflammatory/immune cells in the pathogenesis of pulmonary vascular remodeling. Among inflammatory cell populations, the role of macrophages have been studied extensively in pulmonary vascular remodeling^56^, the results of which suggest that macrophages maintain pulmonary vascular and tissue homeostasis and actively participate in pulmonary vascular remodeling in disease state^56^.

The pathogenic role of macrophages is described in its ability to produce large amounts of pro-inflammatory mediators, which adversely affect vascular cell functions. For example, perivascular macrophages accumulated due to hypoxia exposure releases many inflammatory mediators including interleukin B4^57^, which promotes PAEC apoptosis resulting in capillary rarefaction with subsequent development of PH. The involvement of macrophages in PH is also supported by animal studies in which macrophage deletion or inhibition prevents development of PH^57,58^.

In IPAH and in animal models of PH, it has been shown that NK cells display an impaired phenotype^59^. Further studies revealed that mice deficient for NK cells develop spontaneous PH^60^. Interestingly, Fischer rats develop severe PH with maladaptive RV remodeling in response to SuHx exposure compared with SD rats, and they also display NK cell deficiency in RV tissues^61^. Taken together, these studies suggest that NK cells play a crucial role in maintaining pulmonary vascular and RV homeostasis.

Despite accumulated T cells in the vascular wall in PAH, studies consistently show that circulating T cells are decreased in PAH^42,62^, suggesting the involvement of impaired/ diminished immunity in the pathogenesis of PAH. Interestingly, decreased circulating immune cell populations including NK cells^59,63^ and CD8^+^ T cells^63,64^ have been shown to predict adverse outcome in PAH patients^63^. In line with these observations, animal studies also revealed the important role of T cells in pulmonary vascular homeostasis. For example, athymic nude rats lacking T cells develop severe PH in response to EC apoptosis challenge (SU5416 injection) compared with WT rats, which do not develop PH^65–67^. Accordingly, T cell reconstitution in those rats prevents PH development^66,67^, suggesting the essential role of T cells in maintaining PAEC homeostasis, repair, and proliferation. Interestingly, female athymic rats develop more severe PH compared with male counterparts in response to SU5416 injection^67^, suggesting a more important role of T cells in females.

The beneficial role of T cells in pulmonary vascular remodeling is explained by their ability to inhibit macrophage recruitment to the lung vasculature^68^. In contrast, RAG1^−∕−^ mice lack both B cells and T cells and are protected from monocrotaline-induced PH, which is lost upon the reconstitution of CD4^+^ T cell populations by adoptive transfer^69^. Subsequent reconstitution studies with specific T cell subpopulations in hypoxia-exposed RAG1^−∕−^ mice have revealed that Th17 T cells are responsible for pulmonary vascular remodeling^70^.

Although the mechanisms of T cell accumulation in the pulmonary vessels are not completely understood, available studies indicate that they are recruited secondary to EC damage. For example, studies have revealed that T cell infiltrates perivascular regions to repair EC upon EC damage/apoptosis; however, persistent EC damage leads to the overactivation of T cells, consequently resulting in abnormal PASMC proliferation and vascular remodeling^69^.

Even in the absence of overt inflammatory phenotypes, IPAH patients exhibit mild signs of systemic chronic inflammation, as evidenced by increased circulating cytokines including interleukins^71^ and chemokines^72^. Importantly, a majority of these inflammatory mediators have been found to predict the survival of PAH patients^71,72^.

Currently, there is solid evidence of adverse effects of various cytokines and chemokines on pulmonary vascular cells as shown by pro-apoptotic, pro-proliferative, and pro-migratory roles of different inflammatory mediators on PAECs and PASMCs. In addition, a majority of inflammatory mediators cause PH development in animals and experimental therapeutic strategies targeting them successfully prevent or reverse PH in PH animal models^71,72^. However, targeting the most prominent cytokines such as IL-1^73^ and IL-6^74^ in phase II clinical trials has failed to show effectiveness in PAH patients and resulted in the development of severe side effects, although there are PAH patient subpopulations who benefit from such therapies.

Future clinical trials should consider the heterogeneity of PAH patients to identify PAH subpopulation who could benefit from such therapies. A recent study has shown that PAH patients are highly heterogeneous and can be subcategorized into several inflammatory phenotypes^75^. Thus, further studies are required to better phenotype PAH patients to identify those who may benefit from such strategies.

#### Future perspectives

Taken together, it remains unclear whether deregulated inflammation and immunity is a driver or a consequence of pulmonary vascular remodeling. Current evidence supports inflammation as one of the contributors of disease progression. Nevertheless, preclinical studies have documented the involvement of inflammation and immunity to the pathogenesis of PH with a majority of experimental anti-inflammatory agents being effective in preventing or reversing pulmonary vascular remodeling.

Recent trials have shown that anti-inflammatory strategies are not effective in PAH patients and cause a plethora of side effects. Thus, future studies are required to better understand the role of inflammation in PAH before advancing to clinical trials, and development of novel therapeutic strategies should consider not only suppressing but also restoring the balance and homeostasis of the immune system in PAH.

### V. Deregulated cellular energetics in PH

Deregulation of cellular energetics is not the only the hallmark of cancer, but is necessary for the maintenance of other hallmarks, especially sustaining proliferative signaling, evading growth suppression, and resisting cell death that render highly proliferative features of cancer cells^76^.

Excessive growth increases the demand of proliferative cells for energy and macromolecules. Based on the economic law of supply and demand, in cancer, proliferative cells have to alter their metabolism in a stressful tumor microenvironment where cells have limited accessibility to oxygen and nutrient especially by being far away from blood vessels^77^.

In PH, vessels also show highly proliferative feature in their intima and medial walls that consequently indicate the important role of metabolism as a molecular driver of PH pathogenesis^78^. However, metabolic alterations in PH seem to be more complex than cancers and possess unique features due to the vicinity of proliferative cellular layers of vessel to the bloodstream (the main supplier of nutrients and oxygen).

The reprogrammed metabolism of cancer cells was first discovered by Otto Warburg who indicated that tumor slices and ascites cancer cells constitutively use glucose and produce lactate even in the presence of oxygen^79^. Similar phenomenon was observed in PH vascular cells, i.e., a metabolic shift from glucose oxidation phosphorylation in mitochondria to glycolysis in cytosol^80,81^. However, ATP production (38 ATPs per glucose during oxidative phosphorylation vs 2–4 ATPs per glucose during glycolysis) alone cannot explain the major proliferative advantage gained by cancer cells or pulmonary vascular cells during the metabolic shift from oxidative phosphorylation to glycolysis. Thus, apart from producing ATP, glycolysis also supports ancillary metabolic pathways to enhance proliferation, such as pentose phosphate pathway that yields reductive NADPH and ribose-5-phosphate as the main metabolic blocks for generation of fatty acids and DNA/RNA in proliferating cells^82^. Moreover, lactate production by highly glycolytic proliferative cells not only stimulates angiogenesis as a source of nutrient and oxygen but also induces acidosis in microenvironment, which is toxic for other cells but without harmful effect on proliferating cells because they are armed against apoptosis process^83,84^. Therefore, high glycolytic rate confers selective growth advantages to proliferative cells in both autonomous and nonautonomous manner.

Glycolysis is mainly regulated via 3 signaling pathways: (i) hypoxia-inducible factor (HIF), (ii) phosphatidylinositol 3-kinase (PI3K) and its downstream pathways AKT and mammalian target of rapamycin (mTOR), and (iii) MYC pathway^85^. Among these signaling pathways, HIF is demonstrated to have a significant functional role in both cancer and PH pathogenesis^86^. Furthermore, genetic deficiencies of both HIF-1α and HIF-2α prevented mice from developing hypoxia-induced PH^87^.

HIF is a master hypoxic regulator, participating in cellular adaption to low oxygen level mainly by shifting metabolic responses from oxidation phosphorylation to glycolysis by inducting metabolic genes involved in glycolysis such as pyruvate dehydrogenase kinase (PDK), lactate dehydrogenase (LDH), and glucose transporters (GLUTs)^86^. Under normal oxygen level, HIF is targeted for hydroxylation by prolyl hydroxylase (PHD), thereby marked for final ubiquitination through von-Hippel-Lindau ubiquitin ligase^88^. PHDs belongs to α-ketoglutarate (αKG)-dependent dioxygenase family that uses molecular O_2_ and αKG as substrates and exists in 3 isoforms (PHD1, PHD2, and PHD3)^89^. Thus, PHD-mediated HIF regulation can be modulated by both oxygen dependent and independent (through TCA cycle metabolites) mechanisms. In addition, PHD2 deficiency in PAECs and hematopoietic cells induces obliterative vascular remodeling and severe PH in mice and humans through HIF-2α^90^.

Most importantly, our recent work demonstrated a novel regulatory mechanism of PHD-HIF axis in both PH and cancer. We found that hypoxia-ROS-driven stabilization of a scaffold protein, Ras association domain family 1A (RASSF1A), inhibits HIF-1α proteasomal degradation through blocking its prolyl hydroxylation^91^. PHD-HIF axis regulation is observed not only in hypoxia-induced PH mouse models but also in lung tissues and cell isolated from human IPAH patients, indicating that apart from hypoxia and TCA metabolites can also regulate PHD-HIF axis in PH.

Recent literature indicates that TCA metabolites (αKG, hydroxyglutarate, succinate, etc.) direct a fine-tuned metabolic rewiring by regulating not only HIF but also oxygen-independent glycolysis. Isocitrate dehydrogenase (IDH) converts αKG into isocitrate and has been reported to be increased in the serum of PH patients, which is also derived from EC in patients with *BMPR2* mutation^92^. Therefore, increasing IDH activity can lead to a reduction of αKG and subsequently PHD activity, resulting in HIF stabilization and induction of HIF-associated genes.

Furthermore, 2-hydroxyglutarate (2HG) as a reduced form of αKG is the first discovered oncometabolite. 2HG has 2 isoforms: D2HG is scarce in normal tissues but tumors with IDH1/2 mutations show increase in D2HG concentrations, and L2HG is produced by various dehydrogenases such as LDH and malate dehydrogenase (MDH) but not from mutant IDH1/2. Both isoforms of 2HG have capability to inhibit prolyl hydroxylation of HIF^93^.

In the context of PH, IDH1/2 mutation and consequently D2HG existence are very rare, but human PAECs and PASMCs demonstrated the ability to produce L2HG production upon hypoxic exposure^94^, suggesting that pro-PH factors can induce L2HG production that subsequently inhibits prolyl hydroxylation of HIF and HIF stabilization. Moreover, other TCA metabolites such as succinate and fumarate can also regulate HIF stabilization through modulation of PHD activity^95^.

Loss-of-function mutations in the succinate dehydrogenase (SDH) subunits (mainly SDHB and SDHD) lead to familial paraganglioma (PGL) syndromes characterized in head and neck tumors^96^. PGL patients with SDH mutation showed HIF-2α induction and its downstream associated angiogenic genes expression^97^. Interestingly, the carotid body (CB), the key player in acute adaptation to hypoxia, is the most common tumor site in PGL. It follows that succinate accumulation as a result of SDH-deficient activity and can disturb CB function through modulation of PHD-HIF axis in PG patients. Further investigations are needed to assess whether SDH/succinate activity is involved in PH initiation and development and whether alterations in SDH/succinate activity may induce PH in PG patients.

Based on the pivotal roles of HIF in PH pathogenesis, HIF-targeted therapy can be a potential therapeutic approach in PH patients. It has been shown that PDK level as a HIF-target gene is increased in response to hypoxia and in PH patients. PDK renders a glycolytic phenotype to proliferative cells by inhibiting pyruvate dehydrogenase (PDH) activity, which in turn switches glucose oxidation from TCA cycle to glycolysis. Inhibition of PDK activity by dichloroacetate (DCA) revealed promising outcomes in different PH animal models^98,99^. Targeting other regulatory mechanisms of HIF and the key points of glycolysis can be considered as potential future therapies in PH.

Despite the glycolytic phenotype of malignant and proliferating cells, functional mitochondria are also a unique and general feature for all proliferating cells except those that have mutations in TCA-related genes such as SDH. Proliferating cells use mitochondria to provide the precursors for macromolecule synthesis^100^. However, maintaining the integrity of mitochondrial function is a big challenge for proliferating cells as most of the glucose is used for lactate production instead of acetyl-CoA production for TCA cycle initiation, which is a prerequisite for mitochondrial integrity. Proliferating cells such as cancer cells apply anaplerotic pathways to overcome this problem and to maintain mitochondrial function^101^. Glutaminolysis and pyruvate carboxylation are 2 well-known anaplerotic fluxes that produce αKG from glutamine and oxaloacetate from glucose/pyruvate, respectively^101^.

A large body of evidence identifies the crucial pathogenic roles of glutaminolysis in PH^92,102^. In this regard, mechanical stimuli such as stiff condition have been shown to induce glutaminase 1 (GLS1), resulting in glutaminolysis induction in pulmonary vascular endothelial and SMCs in YAP 1 and the transcriptional coactivator with a PDZ-binding motif (TAZ)-dependent manner. Pharmacological inhibition of YAP or GLS1 also has promising outcomes in rodent PH^103^. The accumulation of glutamate as a GLS1 product is also identified in pulmonary arteries of human PH. This enhancement is associated with stimulation of NMDA-type glutamate receptor, leading to glutamatergic cell-to-cell signaling in pulmonary vessels. Deficiency of NMDAR in smooth muscles in mice and application of pharmacological NMDAR antagonist in rats not only reduce hypoxic vascular remodeling but also diminish proliferation, apoptosis resistance, and especially perivascular inflammation^104^. The immunomodulatory function of glutamine in cancer^105^ suggests that vascular hyperproliferative cells in PH not only replenish their TCA cycle by using glutaminolysis but also can reshape the vascular wall immunophenotypes in a way to provide proliferative/growth advantage.

#### Future perspectives

Although the investigation on PH metabolism has already started, compared with cancer metabolism, there are many untouched areas in the field that have the potential for further exploration (e.g., to explore how altered metabolism can induce apoptosis in vascular cells). Studies suggest that apoptotic cells have distinct metabolic profile. They secrete a specific type of metabolites during cell death that send stimuli to the microenvironment and neighborhood cells to modulate outcomes in the tissue^106^. Therefore, by induction of apoptosis as a treatment approach in PH, we can change the secretory metabolic profile of apoptosis resistant cells, resulting in modulation of genes associated with cytoskeletal rearrangements, inflammation, wound healing, and antiapoptotic functions in other cells especially immune cells such as macrophages^106^. In this regard, apoptosis-induced agents can be combined with specific metabolism-associated therapies in PH to improve the efficiency of apoptosis-based therapies through regulation of the tissue microenvironment.

Given the pivotal roles of metabolism in immune cells function^107^ and the disturbance of immune response in PH^108^, immunometabolism can be a fascinating area to further decipher PH. Succinate and lactate are 2 well-known metabolites with immunomodulatory roles mainly through polarization of macrophages and T cell inactivation^109,110^. Given the high glycolytic rate and distinct mitochondria feature in PH, these 2 metabolites can also regulate immunophenotypes associated with PH. Although abnormalities of NO production are intensively studied in EC during PH in animal models and human PH^111^, the impacts of NO and arginine metabolism disturbances, as 2 regulatory mechanisms of T cell function and activation, on immunophenotypes of PH are not completely understood in PH. Targeting NO/arginine metabolism used in preclinical and clinical trials in PH^111^ can also affect the immunophenotypes of PH, which needs further investigation.

Finally, as previously discussed, HIF can be regulated independent of oxygen level mainly through different metabolites. There is not a precise picture about this type of HIF regulation in PH. Determination of metabolic profile of different cell types in PH especially PAECs and PASMCs during PH initiation and development and associated metabolic pathways can reveal some novel regulatory mechanisms of HIF. Targeting these metabolites and underlying pathways can further increase HIF-dependent therapies in PH.

### VI. Epigenetic mechanisms and transcription factors control hallmarks of PH

Thus far we have largely discussed the critical molecular factors and signaling pathways that are strongly associated with the hallmarks of PAH, which parallels key pathological features of cancer. However, a deeper understanding of the interplay between epigenetic mechanisms and transcription factors (TF) is required to provide a global perspective and to elucidate the molecular programs behind the pathological hallmarks of PAH^112^.

Notably, epigenomic landscape governs DNA accessibility to DNA-binding TFs through multiple mechanisms including histone modifications and DNA methylation, incorporation of histone variants, ATP-dependent chromatin remodeling, and regulation via noncoding RNAs^113^.

Specifically, the involvement of epigenetic mechanisms in PH has been majorly substantiated with the identification of dysregulated proteins from the families of histone deacetylases (HDACs)^114^, sirtuins (SIRTs)^115^, bromodomain-containing proteins (BRDs)^116^, DNA methyltransferases (DNMTs), alterations in DNA methylation^117^, histone H1 levels^118^, histone modifications^119^, and noncoding RNAs^120^.

Markedly, global methylation profiling revealed 147 differential methylated promoters in pulmonary EC (PAEC), including genes involved in cholesterol and lipid transport pathway in PAH^121^. Further, hypermethylation of CpG islands at SOD2 locus was identified in patients with IPAH^117^ and also at granulysin (GNLY) locus in patients with pulmonary veno-occlusive disease, but not in IPAH or heritable PAH^62^. These studies affirm the dysregulation of regulators associated with DNA methylation pathway in PAH.

Besides DNA methylation, histone modifications tightly regulate chromatin structure and DNA accessibility to DNA-binding factors^122^. Different studies have identified increased abundance of HDAC1, HDAC2, and HDAC3, HDAC5 protein levels and HDAC activity in PH-fibroblasts, PAH-PASMCs, and human IPAH lung homogenates. Furthermore, numerous studies have preclinically evaluated the therapeutic potential of commercially available broad spectrum, class-selective HDAC inhibitors in multiple rodent models of PH, as previously reviewed^114^. In addition to their anticancer activity and beneficial effects in preclinical models of left ventricular dysfunction, several broad spectrum, class I/II-specific HDAC inhibitors and SIRT1 activators have been evaluated and reported to mitigated established PH in rodents in vivo.

Regarding attenuation of multiple hallmarks of PAH, several small-molecule compounds inhibited the high proliferative phenotype, migratory capacity, reduced resistance to apoptosis, and inflammatory gene expression in vascular cells^114^. The crucial role of histone acetylation pathway in PAH pathogenesis has been strengthened through the recent report of global alterations in the histone acetylation state at transcriptional enhancers in PAECs isolated from lungs in patients with PAH^119^.

Besides writers (HATs) and erasers (HDACs) of histone acetylation, key reader protein BRD4 was identified to be upregulated in lungs, distal pulmonary arteries, and PASMCs of patients with PAH^116,123^. Proactively, a preclinical multicenter study showed that the clinically available BET inhibitor RVX-208 (Apabetalone) demonstrated to reverse vascular remodeling and improve pulmonary hemodynamics in 2 independent trials in the models of Sugen5416 + hypoxia-PAH and in monocrotaline + shunt-PAH^123^. The authors also demonstrated that RVX208 could be combined safely with current PAH therapies. Furthermore, a clinical trial has been designed to investigate the safety, tolerability, and effectiveness of RVX-208 in PAH patients (NCT03655704)^114^.

Transcriptome profiling studies conducted in PAH have confirmed significant transcriptional dysregulation that ultimately leads to aberrant cellular processes and aggravated vascular remodeling processes^124^. To date, extensive research has acknowledged numerous DNA-binding TFs that were not only associated with different hallmarks of PAH, but were found to either significantly suppress (PPARG1, MEF2, FOXO1, TP53, KLF4) or aggravate (TWIST1, SLUG, HIF1A, HIF2A, STAT3, HES5, SMAD9, OCT4, KLF5, LEF1, PPARGC1A, CREB1, SRF, NF-KB/ RELA, NFATC2, NFATC3, GATA6, c-JUN, c-FOS, EGR1, CTNNB1, FOXM1) vascular remodeling in vivo^125^.

As an emerging concept, interplay between multiple TFs and epigenetic regulators is hypothesized to cooperatively regulate transcriptional programs underlying different vascular cell phenotypes associated with the hallmarks of PAH. For instance, hypoxia is one of major stimulus strongly linked to PH development, wherein hypoxia alters proliferative capacity, apoptosis resistance, and migration of vascular cells, through HIF-dependent or HIF-independent mechanisms^126^.

Remarkably, HIF1A and HIF2A not only positively regulated pro-proliferative FOXM1^126^ under hypoxic conditions, but hypoxic exposure was also associated with inactivation of growth suppressors, TP53^127^ or FOXO1^33^. The imbalance between growth-promoting and growth-suppressive TFs under hypoxia may altogether culminate in antiapoptotic and pro-proliferative cellular responses, which are the major hallmarks of PAH pathogenesis.

Additionally, HIF-mediated transactivation of hypoxia-responsive genes is also regulated by epigenetic enzymes at multiple levels. Besides direct acetylation of HIF-α that increases HIF-1α protein stability and modulates HIF transcriptional activity, acetyltransferases P300/CBP recruited by HIF-1α regulate transcription of numerous HIF-target genes^128^. These findings highlight the complex interplay between TF and the chromatin landscape that is yet to be elucidated in the context of multifactorial disease such as human PAH.

#### Future perspectives

Gene expression regulation involves complex interplay between signaling cascades, metabolic pathways, epigenetic mechanisms, and TFs^129^. Given that PH is a multifactorial disease, a wide range of pathological stimuli including shear stress, hypoxia, or inflammation can modulate signaling cascades that eventually converges on epigenetic regulators and stimulus-specific TFs, which may elicit a transcriptome profile that promotes the hallmarks of PAH.

One of the major therapeutic advantages in targeting TFs or coregulators is their function as major downstream effector that governs disease-specific molecular signatures associated with phenotypic alterations in PAH. Importantly, transcriptional outcome is moderated by sequence-specific TFs, which bind to regulatory DNA elements such as promoters and enhancers and mediate long-range interactions between these elements^130^. A recent study demonstrated alterations in histone acetylation and remodeling of active endothelial enhancers may underlie the aberrant transcriptional responses associated with disturbed angiogenesis and endothelial-to-mesenchymal-transition in PAH-PAECs^119^. Moreover, multiple lines of evidence confirm the existence of significant dysregulation of histone acetylation pathway in PAH.

In a translational perspective, the proteins involved in histone acetylation pathways (HDAC, SIRT, and BRD) still continue to be prospective candidates, as their expression and consequently histone acetylation are significantly altered in PAH^114,131^. Nevertheless, in addition to therapeutic evaluation of isoform-selective small-molecule compounds in achieving reverse remodeling, the vascular cell-specific roles and the spectrum of molecular interactions associated with the dysregulated transcriptional regulators have to be explored in detail. Particularly, high-throughput next-generation sequencing, systems, and network medicine approaches should be employed to attain a global perspective of the disease-specific molecular networks underlying PAH pathogenesis^132,133^.

## Concluding remarks

An intriguing vision of PH as a disease with a cancer-like nature provided unprecedented opportunities for a new understanding of PH and may offer new approaches for PH treatment. Despite striking similarities in PH and cancers, there are fundamental differences that need to be considered for the development of pharmacologic treatments. Cancer treatments have the goal to limit or prevent tumor growth and induce cell death. However, interventions targeting PH aim to reverse structural remodeling and improve endothelial regeneration. In addition, PH is caused by cancer and its therapeutic agents including chemotherapy, radiotherapy, and even the targeted therapies should be taken into consideration.

Tumoral PH includes pulmonary macroembolism and tumors that involve the proximal pulmonary vasculature, such as angiosarcoma; both may mimic pulmonary embolism and chronic thromboembolic PH^134^. Recently, our group has provided clinical, and histopathological evidence of PH in 25% patients with lung cancer. Importantly, we have demonstrated that not the emboli, but rather with microenvironmental inflammation and its cross talk with vascular cells as the major underlying pathogenic pathway.

Pulmonary vascular abnormalities may thus contribute to the symptoms presented by lung cancer patients^135^. This constellation of symptoms will become even more relevant as recently developed therapeutics increase the life expectancy of lung cancer patients.

Finally, tumoral PH may develop in response to treatments of an underlying malignancy. There is increasing interest in PAH induced by tyrosine kinase inhibitors, such as dasatinib. In addition, radiotherapy and chemotherapeutic agents such as mitomycin-C can cause PVOD^134^. Thus, great caution has to be taken to potentially apply some of these cancer therapeutic agents in the cure of PH. In short, the relationship between cancer, cancer therapy, and PH is an interesting one requiring further attention, and research. Examination of these relationships may inspire development of pharmacologic strategies that aim to treat both the cancers and the PH.

## References

[1] Simonneau G (2019). Haemodynamic definitions and updated clinical classification of pulmonary hypertension. Eur Respir J.

[2] Morrell NW (2019). Genetics and genomics of pulmonary arterial hypertension. Eur Respir J.

[3] Humbert M (2019). Pathology and pathobiology of pulmonary hypertension: state of the art and research perspectives. Eur Respir J.

[4] Tuder RM (2013). Relevant issues in the pathology and pathobiology of pulmonary hypertension. J Am Coll Cardiol.

[5] Pullamsetti SS (2017). Translational advances in the field of pulmonary hypertension. From cancer biology to new pulmonary arterial hypertension therapeutics. Targeting cell growth and proliferation signaling hubs. Am J Respir Crit Care Med.

[6] Ghofrani HA (2013). Riociguat for the treatment of chronic thromboembolic pulmonary hypertension. N Engl J Med.

[7] Ghofrani HA (2013). Riociguat for the treatment of pulmonary arterial hypertension. N Engl J Med.

[8] Rai PR (2008). The cancer paradigm of severe pulmonary arterial hypertension. Am J Respir Crit Care Med.

[9] Cool CD (2020). The hallmarks of severe pulmonary arterial hypertension: the cancer hypothesis - ten years later. Am J Physiol Lung Cell Mol Physiol.

[10] Boucherat O (2017). The cancer theory of pulmonary arterial hypertension. Pulm Circ.

[11] Spiekerkoetter E (2019). Hot topics in the mechanisms of pulmonary arterial hypertension disease: cancer-like pathobiology, the role of the adventitia, systemic involvement, and right ventricular failure. Pulm Circ.

[12] Schermuly RT (2005). Reversal of experimental pulmonary hypertension by PDGF inhibition. J Clin Invest.

[13] Sun M (2016). Smooth muscle insulin-like growth factor-1 mediates hypoxia-induced pulmonary hypertension in neonatal mice. Am J Respir Cell Mol Biol.

[14] Tuder RM (2001). Expression of angiogenesis-related molecules in plexiform lesions in severe pulmonary hypertension: evidence for a process of disordered angiogenesis. J Pathol.

[15] Merklinger SL (2005). Epidermal growth factor receptor blockade mediates smooth muscle cell apoptosis and improves survival in rats with pulmonary hypertension. Circulation.

[16] Izikki M (2009). Endothelial-derived FGF2 contributes to the progression of pulmonary hypertension in humans and rodents. J Clin Invest.

[17] Voelkel NF (1998). Primary pulmonary hypertension between inflammation and cancer. Chest.

[18] Yung LM (2016). A selective transforming growth factor-beta ligand trap attenuates pulmonary hypertension. Am J Respir Crit Care Med.

[19] Salmon RM (2020). Molecular basis of ALK1-mediated signalling by BMP9/BMP10 and their prodomain-bound forms. Nat Commun.

[20] Long L (2015). Selective enhancement of endothelial BMPR-II with BMP9 reverses pulmonary arterial hypertension. Nat Med.

[21] Knudsen ES, Wang JY (2010). Targeting the RB-pathway in cancer therapy. Clin Cancer Res.

[22] Cancer Genome Atlas Research, N (2008). Comprehensive genomic characterization defines human glioblastoma genes and core pathways. Nature.

[23] Roskoski Jr R (2016). Cyclin-dependent protein kinase inhibitors including palbociclib as anticancer drugs. Pharmacol Res.

[24] Weiss A (2019). Targeting cyclin-dependent kinases for the treatment of pulmonary arterial hypertension. Nat Commun.

[25] Dillon LM, Miller TW (2014). Therapeutic targeting of cancers with loss of PTEN function. Curr Drug Targets.

[26] Ravi Y (2013). Dysregulation of PTEN in cardiopulmonary vascular remodeling induced by pulmonary hypertension. Cell Biochem Biophys.

[27] Ha D (2014). Pulmonary arterial hypertension in a patient with Cowden syndrome and the PTEN mutation. Pulm Circ.

[28] Nemenoff RA (2008). Targeted deletion of PTEN in smooth muscle cells results in vascular remodeling and recruitment of progenitor cells through induction of stromal cell-derived factor-1alpha. Circ Res.

[29] Oudit GY, Penninger JM (2009). Cardiac regulation by phosphoinositide 3-kinases and PTEN. Cardiovasc Res.

[30] Johnson R, Halder G (2014). The two faces of Hippo: targeting the Hippo pathway for regenerative medicine and cancer treatment. Nat Rev Drug Discov.

[31] Kudryashova TV (2016). HIPPO-integrin-linked kinase cross-talk controls self-sustaining proliferation and survival in pulmonary hypertension. Am J Respir Crit Care Med.

[32] Yan D (2019). Angiotensin-converting enzyme 2 activation suppresses pulmonary vascular remodeling by inducing apoptosis through the Hippo signaling pathway in rats with pulmonary arterial hypertension. Clin Exp Hypertens.

[33] Savai R (2014). Pro-proliferative and inflammatory signaling converge on FoxO1 transcription factor in pulmonary hypertension. Nat Med.

[34] Dabral S (2016). Notch1 signalling regulates endothelial proliferation and apoptosis in pulmonary arterial hypertension. Eur Respir J.

[35] Fan Z (2015). YM155, a selective survivin inhibitor, reverses chronic hypoxic pulmonary hypertension in rats via upregulating voltage-gated potassium channels. Clin Exp Hypertens.

[36] He C, Lu J, Lin W (2015). Hybrid nanoparticles for combination therapy of cancer. J Control Release.

[37] Ghofrani HA, Seeger W, Grimminger F (2005). Imatinib for the treatment of pulmonary arterial hypertension. N Engl J Med.

[38] Ghofrani HA (2010). Imatinib in pulmonary arterial hypertension patients with inadequate response to established therapy. Am J Respir Crit Care Med.

[39] Savai R (2012). Immune and inflammatory cell involvement in the pathology of idiopathic pulmonary arterial hypertension. Am J Respir Crit Care Med.

[40] Tuder RM (1994). Exuberant endothelial cell growth and elements of inflammation are present in plexiform lesions of pulmonary hypertension. Am J Pathol.

[41] Stacher E (2012). Modern age pathology of pulmonary arterial hypertension. American journal of respiratory and critical care medicine.

[42] Perros F (2012). Pulmonary lymphoid neogenesis in idiopathic pulmonary arterial hypertension. Am J Respir Crit Care Med.

[43] Nicolls MR (2005). Autoimmunity and pulmonary hypertension: a perspective. Eur Respir J.

[44] Nicolls M (2005). Autoimmunity and pulmonary hypertension: a perspective. European Respiratory Journal.

[45] Isern RA (1992). Autoantibodies in patients with primary pulmonary hypertension: association with anti-Ku. Am J Med.

[46] Rich S (1986). Antinuclear antibodies in primary pulmonary hypertension. J Am Coll Cardiol.

[47] Tamby MC (2005). Anti-endothelial cell antibodies in idiopathic and systemic sclerosis associated pulmonary arterial hypertension. Thorax.

[48] Terrier B (2008). Identification of target antigens of antifibroblast antibodies in pulmonary arterial hypertension. Am J Respir Crit Care Med.

[49] Kherbeck N (2013). The role of inflammation and autoimmunity in the pathophysiology of pulmonary arterial hypertension. Clinical reviews in allergy & immunology.

[50] Bussone G (2012). IgG from patients with pulmonary arterial hypertension and/or systemic sclerosis binds to vascular smooth muscle cells and induces cell contraction. Annals of the rheumatic diseases.

[51] Tamby MC (2006). Antibodies to fibroblasts in idiopathic and scleroderma-associated pulmonary hypertension. European Respiratory Journal.

[52] Basyal B, Jarrett H, Barnett CF (2019). Pulmonary hypertension in HIV. Canadian Journal of Cardiology.

[53] Fernández AI (2019). The biological bases of group 2 pulmonary hypertension. International journal of molecular sciences.

[54] Gredic M (2020). Pulmonary hypertension in chronic obstructive pulmonary disease. Br J Pharmacol.

[55] Koudstaal T, Boomars KA, Kool M (2020). Pulmonary arterial hypertension and chronic thromboembolic pulmonary hypertension: an immunological perspective. Journal of clinical medicine.

[56] Florentin J, Dutta P (2017). Origin and production of inflammatory perivascular macrophages in pulmonary hypertension. Cytokine.

[57] Tian W (2013). Blocking macrophage leukotriene b4 prevents endothelial injury and reverses pulmonary hypertension. Science translational medicine.

[58] Thenappan T (2011). A central role for CD68 (+) macrophages in hepatopulmonary syndrome: reversal by macrophage depletion. American journal of respiratory and critical care medicine.

[59] Ormiston ML (2012). Impaired natural killer cell phenotype and function in idiopathic and heritable pulmonary arterial hypertension. Circulation.

[60] Ratsep MT (2018). Spontaneous pulmonary hypertension in genetic mouse models of natural killer cell deficiency. Am J Physiol Lung Cell Mol Physiol.

[61] Suen CM (2019). Fischer rats exhibit maladaptive structural and molecular right ventricular remodelling in severe pulmonary hypertension: a genetically prone model for right heart failure. Cardiovasc Res.

[62] Perros F (2013). Cytotoxic cells and granulysin in pulmonary arterial hypertension and pulmonary veno-occlusive disease. Am J Respir Crit Care Med.

[63] Edwards AL (2013). Professional killer cell deficiencies and decreased survival in pulmonary arterial hypertension. Respirology.

[64] Ulrich S (2008). Increased regulatory and decreased CD8+ cytotoxic T cells in the blood of patients with idiopathic pulmonary arterial hypertension. Respiration.

[65] Taraseviciene-Stewart L (2007). Absence of T cells confers increased pulmonary arterial hypertension and vascular remodeling. American journal of respiratory and critical care medicine.

[66] Tamosiuniene R (2011). Regulatory T cells limit vascular endothelial injury and prevent pulmonary hypertension. Circ Res.

[67] Tamosiuniene R (2018). Dominant Role for Regulatory T Cells in Protecting Females Against Pulmonary Hypertension. Circ Res.

[68] Gerasimovskaya E (2012). Interplay of macrophages and T cells in the lung vasculature. Am J Physiol Lung Cell Mol Physiol.

[69] Cuttica MJ (2011). Perivascular T-cell infiltration leads to sustained pulmonary artery remodeling after endothelial cell damage. Am J Respir Cell Mol Biol.

[70] Maston LD (2017). Central role of T helper 17 cells in chronic hypoxia-induced pulmonary hypertension. Am J Physiol Lung Cell Mol Physiol.

[71] Rabinovitch M (2014). Inflammation and immunity in the pathogenesis of pulmonary arterial hypertension. Circulation research.

[72] Mamazhakypov A (2019). The role of chemokines and chemokine receptors in pulmonary arterial hypertension. Br J Pharmacol.

[73] Trankle CR (2019). IL-1 blockade reduces inflammation in pulmonary arterial hypertension and right ventricular failure: a single-arm. open-label, phase IB/II pilot study. American journal of respiratory and critical care medicine.

[74] Toshner M (2018). Transform-UK: a phase 2 trial of tocilizumab in pulmonary arterial hypertension, in D108. Good vibrations: Novel treatment approaches in pulmonary hypertension.

[75] Sweatt AJ (2019). Discovery of Distinct Immune Phenotypes Using Machine Learning in Pulmonary Arterial Hypertension. Circ Res.

[76] Hanahan D, Weinberg RA (2011). Hallmarks of cancer: the next generation. Cell.

[77] Brown JM, Giaccia AJ (1998). The unique physiology of solid tumors: opportunities (and problems) for cancer therapy. Cancer Res.

[78] Gurtu V, Michelakis ED (2015). Emerging therapies and future directions in pulmonary arterial hypertension. Can J Cardiol.

[79] Koppenol WH, Bounds PL, Dang CV (2011). Otto Warburg’s contributions to current concepts of cancer metabolism. Nat Rev Cancer.

[80] Sutendra G (2011). The role of Nogo and the mitochondria-endoplasmic reticulum unit in pulmonary hypertension. Sci Transl Med.

[81] Sutendra G (2011). Pyruvate dehydrogenase inhibition by the inflammatory cytokine TNFalpha contributes to the pathogenesis of pulmonary arterial hypertension. J Mol Med (Berl).

[82] Lunt SY, Vander Heiden MG (2011). Aerobic glycolysis: meeting the metabolic requirements of cell proliferation. Annu Rev Cell Dev Biol.

[83] Gatenby RA, Gillies RJ (2004). Why do cancers have high aerobic glycolysis?. Nat Rev Cancer.

[84] Beckert S (2006). Lactate stimulates endothelial cell migration. Wound Repair Regen.

[85] Ward PS, Thompson CB (2012). Signaling in control of cell growth and metabolism. Cold Spring Harb Perspect Biol.

[86] Shimoda LA, Semenza GL (2011). HIF and the lung: role of hypoxia-inducible factors in pulmonary development and disease. Am J Respir Crit Care Med.

[87] Dai Z (2016). Prolyl-4 hydroxylase 2 (PHD2) deficiency in endothelial cells and hematopoietic cells induces obliterative vascular remodeling and severe pulmonary arterial hypertension in mice and humans through hypoxia-inducible factor-2alpha. Circulation.

[88] Huang LE (1998). Regulation of hypoxia-inducible factor 1alpha is mediated by an O2-dependent degradation domain via the ubiquitin-proteasome pathway. Proc Natl Acad Sci U S A.

[89] Loenarz C, Schofield CJ (2008). Expanding chemical biology of 2-oxoglutarate oxygenases. Nat Chem Biol.

[90] Semenza GL, HIF-1 O(2) (2001). HIF-1, O(2), and the 3 PHDs: how animal cells signal hypoxia to the nucleus. Cell.

[91] Dabral S (2019). A RASSF1A-HIF1alpha loop drives Warburg effect in cancer and pulmonary hypertension. Nat Commun.

[92] Fessel JP (2012). Metabolomic analysis of bone morphogenetic protein receptor type 2 mutations in human pulmonary endothelium reveals widespread metabolic reprogramming. Pulm Circ.

[93] Xu W (2011). Oncometabolite 2-hydroxyglutarate is a competitive inhibitor of alpha-ketoglutarate-dependent dioxygenases. Cancer Cell.

[94] Oldham WM (2015). Hypoxia-mediated increases in L-2-hydroxyglutarate coordinate the metabolic response to reductive stress. Cell Metab.

[95] Bailey PSJ, Nathan JA (2018). Metabolic regulation of hypoxia-inducible transcription factors: the role of small molecule metabolites and iron. Biomedicines.

[96] Baysal BE (2000). Mutations in SDHD, a mitochondrial complex II gene, in hereditary paraganglioma. Science.

[97] Gimenez-Roqueplo AP (2001). The R22X mutation of the SDHD gene in hereditary paraganglioma abolishes the enzymatic activity of complex II in the mitochondrial respiratory chain and activates the hypoxia pathway. Am J Hum Genet.

[98] Zhang S (2014). The pivotal role of pyruvate dehydrogenase kinases in metabolic flexibility. Nutr Metab (Lond).

[99] Michelakis ED (2017). Inhibition of pyruvate dehydrogenase kinase improves pulmonary arterial hypertension in genetically susceptible patients. Sci Transl Med.

[100] Ahn CS, Metallo CM (2015). Mitochondria as biosynthetic factories for cancer proliferation. Cancer Metab.

[101] DeBerardinis RJ, Chandel NS (2016). Fundamentals of cancer metabolism. Sci Adv.

[102] Piao L (2013). Cardiac glutaminolysis: a maladaptive cancer metabolism pathway in the right ventricle in pulmonary hypertension. J Mol Med (Berl).

[103] Bertero T (2016). Vascular stiffness mechanoactivates YAP/TAZ-dependent glutaminolysis to drive pulmonary hypertension. J Clin Invest.

[104] Dumas SJ (2018). NMDA-type glutamate receptor activation promotes vascular remodeling and pulmonary arterial hypertension. Circulation.

[105] Palmieri EM (2017). Pharmacologic or genetic targeting of glutamine synthetase skews macrophages toward an M1-like phenotype and inhibits tumor metastasis. Cell Rep.

[106] Medina CB (2020). Metabolites released from apoptotic cells act as tissue messengers. Nature.

[107] Pearce EL, Pearce EJ (2013). Metabolic pathways in immune cell activation and quiescence. Immunity.

[108] Kumar R, Graham B (2018). How does inflammation contribute to pulmonary hypertension?. Eur Respir J.

[109] Pucino V (2019). Lactate buildup at the site of chronic inflammation promotes disease by inducing CD4(+) T cell metabolic rewiring. Cell Metab.

[110] Wu JY (2020). Cancer-derived succinate promotes macrophage polarization and cancer metastasis via succinate receptor. Mol Cell.

[111] Klinger JR, Abman SH, Gladwin MT (2013). Nitric oxide deficiency and endothelial dysfunction in pulmonary arterial hypertension. Am J Respir Crit Care Med.

[112] Chelladurai P, Seeger W, Pullamsetti SS (2016). Epigenetic mechanisms in pulmonary arterial hypertension: the need for global perspectives. Eur Respir Rev.

[113] Li B, Carey M, Workman JL (2007). The role of chromatin during transcription. Cell.

[114] Chelladurai P (2019). Targeting histone acetylation in pulmonary hypertension and right ventricular hypertrophy. Br J Pharmacol.

[115] Zurlo G (2018). Sirtuin 1 regulates pulmonary artery smooth muscle cell proliferation: role in pulmonary arterial hypertension. J Hypertens.

[116] Meloche J (2015). Bromodomain-containing protein 4: the epigenetic origin of pulmonary arterial hypertension. Circ Res.

[117] Archer SL (2010). Epigenetic attenuation of mitochondrial superoxide dismutase 2 in pulmonary arterial hypertension: a basis for excessive cell proliferation and a new therapeutic target. Circulation.

[118] Talati M (2012). Altered expression of nuclear and cytoplasmic histone H1 in pulmonary artery and pulmonary artery smooth muscle cells in patients with IPAH. Pulm Circ.

[119] Reyes-Palomares A (2020). Remodeling of active endothelial enhancers is associated with aberrant gene-regulatory networks in pulmonary arterial hypertension. Nat Commun.

[120] Kim JD (2015). Epigenetic modulation as a therapeutic approach for pulmonary arterial hypertension. Exp Mol Med.

[121] Hautefort A (2017). Pulmonary endothelial cell DNA methylation signature in pulmonary arterial hypertension. Oncotarget.

[122] Cohen I (2011). Histone modifiers in cancer: friends or foes?. Genes Cancer.

[123] Van der Feen DE (2019). Multicenter preclinical validation of BET inhibition for the treatment of pulmonary arterial hypertension. Am J Respir Crit Care Med.

[124] Rhodes CJ (2015). RNA sequencing analysis detection of a novel pathway of endothelial dysfunction in pulmonary arterial hypertension. Am J Respir Crit Care Med.

[125] Pullamsetti SS (2016). Transcription factors, transcriptional coregulators, and epigenetic modulation in the control of pulmonary vascular cell phenotype: therapeutic implications for pulmonary hypertension (2015 Grover Conference series). Pulm Circ.

[126] Raghavan A (2012). Hypoxia-induced pulmonary arterial smooth muscle cell proliferation is controlled by forkhead box M1. Am J Respir Cell Mol Biol.

[127] Wang Z (2019). Divergent changes of p53 in pulmonary arterial endothelial and smooth muscle cells involved in the development of pulmonary hypertension. Am J Physiol Lung Cell Mol Physiol.

[128] Luo W, Wang Y (2018). Epigenetic regulators: multifunctional proteins modulating hypoxia-inducible factor-alpha protein stability and activity. Cell Mol Life Sci.

[129] Ahsendorf T (2017). Transcription factors, coregulators, and epigenetic marks are linearly correlated and highly redundant. PLoS One.

[130] Palstra RJ, Grosveld F (2012). Transcription factor binding at enhancers: shaping a genomic regulatory landscape in flux. Front Genet.

[131] Li P, Ge J, Li H (2020). Lysine acetyltransferases and lysine deacetylases as targets for cardiovascular disease. Nat Rev Cardiol.

[132] Leopold JA, Maron BA, Loscalzo J (2020). The application of big data to cardiovascular disease: paths to precision medicine. J Clin Invest.

[133] Kurnat-Thoma E (2020). Recent advances in systems and network medicine: meeting report from the first international conference in systems and network medicine. Syst Med (New Rochelle).

[134] Price LC (2019). Tumoral pulmonary hypertension. Eur Respir Rev.

[135] Pullamsetti SS (2017). Lung cancer-associated pulmonary hypertension: Role of microenvironmental inflammation based on tumor cell-immune cell cross-talk. Sci Transl Med.

